# Factors influencing referrals for ultrasound-diagnosed complications during prenatal care in five low and middle income countries

**DOI:** 10.1186/s12978-018-0647-8

**Published:** 2018-12-12

**Authors:** Holly L. Franklin, Waseem Mirza, David L. Swanson, Jamie E. Newman, Robert L. Goldenberg, David Muyodi, Lester Figueroa, Robert O. Nathan, Jonathan O. Swanson, Nicole Goldsmith, Nancy Kanaiza, Farnaz Naqvi, Irma Sayury Pineda, Walter López-Gomez, Dorothy Hamsumonde, Victor Lokomba Bolamba, Elizabeth V. Fogleman, Sarah Saleem, Fabian Esamai, Edward A. Liechty, Ana L. Garces, Nancy F. Krebs, K. Michael Hambidge, Elwyn Chomba, Musaku Mwenechanya, Waldemar A. Carlo, Antoinette Tshefu, Adrien Lokangaka, Carl L. Bose, Marion Koso-Thomas, Menachem Miodovnik, Elizabeth M. McClure

**Affiliations:** 1 RTI International, Research Triangle Park, NC USA; 2 Aga Khan University, Karachi, Pakistan; 3 University of Washington, Seattle, WA USA; 4 Columbia University, New York, NY USA; 5 Moi University, Eldoret, Kenya; 6 INCAP, Guatemala City, Guatemala; 7 University of Zambia Lusaka, Lusaka, Zambia; 8 Kinshasa School of Public Health, Kinshasa, Democratic Republic of the Congo; 9 University of Colorado, Denver, CO USA; 10 Indiana University, Indianapolis, IN USA; 11 University of Alabama at Birmingham, Birmingham, AL UK; 12 University of North Carolina at Chapel Hill, Chapel Hill, NC USA; 13 NICHD, Bethesda, MD USA

**Keywords:** Ultrasound, Antenatal care, Low-middle income countries, Pregnancy complication, Hospital referral, Delivery

## Abstract

**Background:**

Ultrasound during antenatal care (ANC) is proposed as a strategy for increasing hospital deliveries for complicated pregnancies and improving maternal, fetal, and neonatal outcomes. The First Look study was a cluster-randomized trial conducted in the Democratic Republic of Congo, Guatemala, Kenya, Pakistan and Zambia to evaluate the impact of ANC-ultrasound on these outcomes. An additional survey was conducted to identify factors influencing women with complicated pregnancies to attend referrals for additional care.

**Methods:**

Women who received referral due to ANC ultrasound findings participated in structured interviews to characterize their experiences. Cochran-Mantel-Haenszel statistics were used to examine differences between women who attended the referral and women who did not. Sonographers’ exam findings were compared to referred women’s recall.

**Results:**

Among 700 referred women, 510 (71%) attended the referral. Among referred women, 97% received a referral card to present at the hospital, 91% were told where to go in the hospital, and 64% were told that the hospital was expecting them. The referred women who were told who to see at the hospital (88% vs 66%), where to go (94% vs 82%), or what should happen, were more likely to attend their referral (68% vs 56%). Barriers to attending referrals were cost, transportation, and distance. Barriers after reaching the hospital were substantial. These included not connecting with an appropriate provider, not knowing where to go, and being told to return later. These barriers at the hospital often led to an unsuccessful referral.

**Conclusions:**

Our study found that ultrasound screening at ANC alone does not adequately address barriers to referrals. Better communication between the sonographer and the patient increases the likelihood of a completed referral. These types of communication include describing the ultrasound findings, including the reason for the referral, to the mother and staff; providing a referral card; describing where to go in the hospital; and explaining the procedures at the hospital. Thus, there are three levels of communication that need to be addressed to increase completion of appropriate referrals-communication between the sonographer and the woman, the sonographer and the clinic staff, and the sonographer and the hospital.

**Trial registration:**

NCT01990625.

## Plain English summary

Ultrasound during antenatal care is proposed as a strategy for increasing hospital deliveries for complicated pregnancies and improving mother and baby outcomes in low and middle-income countries. The First Look study was a trial conducted in the Democratic Republic of Congo, Guatemala, Kenya, Pakistan and Zambia to evaluate the impact of ANCultrasound on pregnancy outcomes. During the study, fewer women than expected completed referrals for additional care. To investigate the reasons for this, structured interviews were administered during the trial to participants who received an ultrasound that identified a possible pregnancy complication. The women were referred by study-trained sonographers based at primary healthcare centers.

The responses from 700 women helped characterize positive and negative reasons that influenced decision making. Cochran-Mantel-Haenszel statistics were used to examine differences between women who attended the referral and women who did not. Sonographers’ findings were compared to women’s recall.

The results indicate that communication between the sonographer and the patient increases the likelihood of a completed referral. Communication includes describing the ultrasound findings and reason for the referral, describing where to go in the hospital, and explaining what to expect at the hospital. Women also encountered barriers after reaching the hospital. These barriers included not seeing an appropriate provider and being told to return later. There are three levels of communication that need to be addressed to promote appropriate referrals - sharing information between the sonographer and the woman, the sonographer and the clinic staff, and the sonographer and the hospital.

## Background

Maternal, fetal, and newborn mortality remains high in low-middle income countries (LMIC). Increasing access to care for women’s pregnancy complications in low-resource settings can improve pregnancy outcomes [1]. Development of evidence-based approaches to improving access to skilled care at birth, timely comprehensive emergency obstetric care, and immediate newborn care in these settings is broadly recommended [2]. Further research into approaches that identify women with high-risk pregnancies and encourage them to deliver in hospitals providing comprehensive emergency obstetric and neonatal care is called for in the literature [3].

In high-income countries, obstetric ultrasound is used routinely at antenatal care (ANC) to determine accurate gestational age and to screen for pregnancy complications [4]. Some LMIC studies have suggested that introduction of ultrasound use during ANC has the potential to increase appropriate referrals for delivery and can lead to reductions in mortality [5–14].

We conducted the First Look trial to assess the impact of ANC ultrasound on pregnancy outcomes in women residing in LMIC [15]. The study trained healthcare personnel based at primary health centers to conduct basic obstetric ultrasound screening and to refer women identified with potentially complicated pregnancies to higher levels of ANC. When the study monitoring data were reviewed, it became clear that attendance of referrals did not occur as expected. The study results showed that hospital deliveries did not increase, and that maternal, neonatal, and fetal outcomes did not improve [16]. To further explore why women with potentially complicated pregnancies diagnosed by ultrasound either followed through or did not follow through on referrals to higher levels of healthcare after the encouragement of study-trained sonographers equipped with ultrasound findings, we conducted structured interviews of women who received such referrals. We sought to understand women’s knowledge and behaviors related to ultrasound screening and subsequent referral attendance. Specifically, we sought to identify barriers to attending referral visits, as well as the motivations for attending the referral. We highlight below the insights provided from this study and discuss the possible implications for widespread ultrasound use at ANC visits in LMIC.

## Methods

The First Look trial was a cluster-randomized trial conducted in rural areas of the Democratic Republic of the Congo (DRC), Guatemala, Kenya, Pakistan, and Zambia to evaluate the impact of basic obstetric ultrasound provided at ANC on maternal, fetal, and neonatal mortality. The study design, including definition of the clusters and the ultrasound training of the sonographers, is described in detail elsewhere [15–17]. The First Look Trial used primary outcome data collected via a health registry that collects antenatal, prenatal, maternal, fetal, and neonatal data on all deliveries in the study intervention and control clusters at three time points: 1) screening and enrollment, 2) delivery, and 3) six weeks post-partum [18]. For the study, 58 geographic areas or clusters each with a central health center responsible for 300 to 500 pregnancies per year were randomized to be an intervention site or to receive usual care. Women residing in the intervention clusters were invited to receive an obstetric ultrasound at 16 to 22 weeks and again at 32 to 36 weeks.

The study-trained sonographers offered ultrasound exams to all pregnant women residing in the study intervention clusters who attended ANC and 77.6% had at least one study examination [16]. The sonographers were trained to assess gestational age and identify potential high-risk pregnancies, including multiple gestations, fetal anomalies, mal-presentations, placenta previa, oligohydramnios, polyhydramnios, intrauterine growth restriction (IUGR), and cervical insufficiency. They were also trained to show the women what they saw on the ultrasound screen, and communicate their findings directly to the patients, their families when available, and other health center providers, to ensure they understood why and when patients should attend referral and/or deliver at the hospital. Referral to higher levels of care was recommended per country guidelines for women with potentially high-risk pregnancies [15]. We emphasize that the referral hospitals were often the only facility within a reasonable distance that would accept clinic referrals. We have studied many of these hospitals previously and documented the level of care available and noted issues such as inadequate medications, equipment and staffing among other issues [19].

Referral algorithms, which were customized with local health system input for each site, provided guidance on the need and the timing of referrals according to the screening results. When screening indicated a potentially high-risk pregnancy, these findings required confirmation by a hospital sonographer and/or an ultrasound-competent physician [17]. If the findings of a high-risk pregnancy were confirmed at the hospital, women were instructed by hospital staff to return to the hospital around the time of delivery.

Structured interviews were conducted during the second half of the trial among a convenience sample of study participants who had received referrals to better understand the barriers and motivators for attending a referral visit. A secondary objective was to measure the women’s understanding of why they were referred. Study staff at each of the five country sites approached up to 200 women from intervention clusters who had received a referral. These women were invited to complete this study’s structured interviews, in addition to their scheduled registry interview, at the 6 weeks post-delivery visit (Table 1) [18]. At this visit, women who had consented to the study, were administered structured interviews by registry staff. Depending on women’s responses to the initial study interview, they were administered one of two additional structured interviews. Women who attended the referral visit were administered an interview designed to identify facilitators that influence attending the referral. Women who did not attend their referral visit were administered a different interview designed to identify potential barriers to attending the referral. The structured interviews collected categorical data using standardized questionnaires.

**Table 1:** Overview of Structured Interview Study Design

Data collected from	*N*	Purpose and Objectives
Up to 200 women per site who received a referral for possible pregnancy complications identified during study ultrasound exam. All of the interviewed women received the initial interview.	700	To determine whether women who received referrals from study sonographers at primary health care centers attended the referrals. They were also interviewed to gain evidence about their understanding of ultrasound and the ultrasound findings/referral instructions, intention to attend the referral visit, expectations of ultrasound, perceptions of potential harm/benefits, experience with receiving the ultrasound, and their recall of the reason for the referral.
Consented women who attended referral visit and agreed to be interviewed at 6-week postpartum study visit scheduled to collect health outcome data.	510	This second interview focused on reasons for attending the referral visit and identified barriers and motivators/facilitators to attending the referral visit.
Consented women who did not attend referral visit and agreed to be interviewed at 6-week postpartum study visit scheduled to collect health outcome data.	190	This second interview focused on reasons for not attending the referral visit and identified barriers and motivators/facilitators to attending the referral visit.

### Data analysis

Each research site securely transmitted data to the study’s central data coordinating center at RTI International (Durham, NC). All analyses were performed using SAS version 9.4.

We used Cochran-Mantel-Haenszel (CMH) statistics to compare maternal baseline characteristics and factors potentially associated with referral visit attendance between women who attended the referral visit and women who did not, while accounting for the clustered structure of the data. We compared the ultrasound findings documented by the sonographer at the examination as reasons for referral with women’s recall of findings by calculating the percent agreement and the first order of agreement coefficient (AC1) [20]. We calculated percent agreement as the ratio of times the sonographer and woman agreed divided by the total number of ratings performed. We calculated percent agreement overall and separately according to referral visit attendance.

### Ethical approvals

Institutional Review Board approval was obtained from the ethics committees at each of the research sites (Aga Khan University, Pakistan; Moi University, Kenya; Instituto de Nutrición de Centroamérica y Panama, Guatemala; University of Zambia, Zambia; and Kinshasa School of Public Health, DRC), Columbia University, and RTI International. Each participant provided informed consent prior to study participation.

## Results

The main study recruited 24,008 women who had at least one ultrasound examination during ANC and 2233 (9.3%) women received at least one referral. Of the 2233 referred women, 1587 (71.1%) attended a referral and 28.9% did not [15, 16]. Our study completed two structured interviews on a total of 700 of these referred women, achieving a diverse sample: 186 were from Guatemala, 176 were from Zambia, 152 were from Pakistan, 143 were from DRC, and 43 were from Kenya. A total of 510 (73%) had attended the referral visit and 190 (27%) did not attend the referral visit (Table 1). More than 90% of the participants in DRC, Kenya, and Zambia attended their referral visit, while just over half of participants in Guatemala (52%) and Pakistan (55%) attended the visit.

Characteristics of women who attended the referral visit and those who did not are presented in Tables 2 and 3. More than three -quarters of both groups were between the ages of 20–35 years, the majority had two or more children, and one-third had completed primary school. We observed differences among the two groups in the number of previous ultrasounds, location of previous deliveries, and whether the woman planned to attend the referral visit (each *p* < 0.001). A higher proportion of women that attended the referral visit reported having three or more previous ultrasounds (78% vs 68%), delivered in a hospital (49% vs 23%) or clinic/health center (35% vs 19%), and planned to attend the referral visit (98% vs 45%) than women who did not attend the referral visit (p < 0.001).

**Table 2:** Characteristics of Women by Referral Attendance Status

Characteristic	*N* (%)		
	Attended Referral *N* = 510	Did Not Attend Referral *N* = 190	*p*-value
Maternal age (years)^a, d^			0.17
< 20	56/510 (11.0)	27/190 (14.2)	
20–35	405/510 (79.4)	147/190 (77.4)	
> 35	49/510 (9.6)	16/190 (8.4)	
Maternal education^a, d^			0.11
No formal schooling	127/510 (24.9)	77/190 (40.5)	
Primary	163/510 (32.0)	69/190 (36.3)	
Secondary	207/510 (40.6)	40/190 (21.1)	
University	13/510 (2.5)	4/190 (2.1)	
Parity^a, b, d^			0.75
0	95/500 (19.0)	33/181 (18.2)	
1	89/500 (17.8)	37/181 (20.4)	
2+	316/500 (63.2)	111/181 (61.3)	
Previous ultrasounds^d^	508	190	<.0001*
1	15/508 (3.0)	11/190 (5.8)	
2	98/508 (19.3)	49/190 (25.8)	
3+	395/508 (77.8)	130/190 (68.4)	
Location of Previous Delivery^c, e^			<.0001*
Hospital	252/510 (49.4)	43/190 (22.6)	
Clinic/Health center	180/510 (35.3)	36/190 (18.9)	
Home in village	77/510 (15.1)	107/190 (56.3)	
Other	1/510 (0.2)	4/190 (2.1)	

^a^Collected at time of entry into Maternal Newborn Health Registry (MNH). [18]

^b^Not including this pregnancy

^c^Collected during delivery while enrolled in Maternal Newborn Health Registry (MNH)

^d^Hypothesis test results: *p*-values calculated from Cochran-Mantel-Haenszel test (ANOVA statistic)

^e^Hypothesis test results: p-values calculated from Cochran-Mantel-Haenszel test (General Association statistic)

*Denotes p-value < 0.05

**Table 3:** Comparison of Women’s Referral Support and Knowledge by Referral Attendance Status

Response	*N* (%)		
	Attended Referral	Did Not Attend Referral	*p*-value
Know other mothers that have had US^a^	452/510 (88.6)	164/190 (86.3)	0.9199
Baby’s father thinks she should have US^a^	486/509 (95.5)	183/190 (96.3)	0.6845
Other family members think she should have US^a^	492/509 (96.7)	179/190 (94.2)	0.0454*
Planned to attend referral visit^a^	493/503 (98.0)	84/186 (45.2)	<.0001*
Told the date that she should go to referral hospital^a^	446/505 (88.3)	163/188 (86.7)	0.0299*
Told where in the hospital to visit first^a^	476/505 (94.3)	154/188 (81.9)	0.0002*
Given a card that indicated she should go to the referral hospital, to show at hospital^a^	486/505 (96.2)	184/188 (97.9)	0.5759
Told who to see at the referral hospital^a^	444/505 (87.9)	122/186 (65.6)	<.0001*
Knew what the process would be once at the hospital^a^	316/503 (62.8)	115/185 (62.2)	0.0143*
Told that hospital is expecting her arrival^a^	344/502 (68.5)	103/185 (55.7)	0.0002*

Abbreviation: *US* ultrasound

^a^Hypotheses test results: p-values calculated from Cochran-Mantel-Haenszel test (General Association statistic)

*Denotes p-value < 0.05

Among the 700 women, 97% said they were given a referral card that they could present at the hospital, 91% said they were told where to go in the hospital, 88% said they were told when to arrive, 82% said they were told who to see at the hospital, and 64% said they were told that the hospital was expecting their arrival. In addition, 62% of the women said they knew what the process would be once they arrived at the hospital.

Observed differences between women who attended a referral visit and those who did not include whether women recalled being told where in the hospital to visit first (94% vs 82%), who to see at the referral hospital (88% vs 66%), and if the hospital was expecting her arrival (67% vs 56%) (p < 0.001). Nearly all women in both groups report being given a card that they could show to the hospital indicating that they had been referred (96% vs 98%); 63% of women who attended the referral visit and 62% of women who did not attend the referral visit indicated they knew what the process would be once at the hospital. A high proportion of women in both groups knew other mothers who had received ultrasounds (89% vs 86%), indicated the baby’s father thought they should have an ultrasound (96% for both groups), and indicated that other family members thought they should have an ultrasound (97% vs 94%).

The most frequent responses concerning expectations of ultrasound, perceptions of potential harms or benefits, and experiences with receiving the ultrasound are detailed in Table 4. A higher proportion of women who attended the referral visit (66%) cited “finding problems that with treatment can help my baby” as a benefit of ultrasound than women who did not attend the referral visit (44%) (*p* < 0.05). The majority in both groups reported benefits of “knowing my baby is doing well” and “seeing my baby.” More than 80% of women reported there to be no harm related to the ultrasound while few women reported that potential harm “made me worry.” Women were asked what they expected to learn before having the ultrasound, and 71% of women who attended the referral visit expected to receive confirmation that the baby was healthy as compared to 54% of women who did not attend the referral visit (p < 0.05). More than 80% of women in both groups indicated they planned to talk to the baby’s father after the ultrasound. Of women who attended the referral visit, 59% indicated they had planned to talk to other family members about the findings compared to 31% of women who did not attend the referral visit (p < 0.05).

**Table 4:** Women’s Expectations and Perceptions by Referral Attendance Status

	*N* (%)		
	Attended Referral *N* = 510	Did Not Attend Referral *N* = 190	*p*-value^a^
Advantages/benefits of US			
Know my baby is doing well	390/510 (76.5)	114/190 (60.0)	0.0817
Seeing my baby	317/510 (62.2)	107/190 (56.3)	0.6594
Find problems that with treatment can help my baby	337/510 (66.1)	84/190 (44.2)	0.0344*
Bad effects/harms of US			
No harms	442/510 (86.7)	152/190 (80.0)	0.8778
Make me worry	26/510 (5.1)	11/190 (5.8)	0.6529
Expected to learn before having US			
Confirmation that the baby is healthy	364/510 (71.4)	102/190 (53.7)	0.0081*
An opportunity to see the baby	285/510 (55.9)	77/190 (40.5)	0.6111
Expected date of delivery	240/510 (47.1)	82/190 (43.2)	0.7512
Additional information would have liked to have BEFORE having US			
Ultrasound is safe for mom and baby	221/510 (43.3)	73/190 (38.4)	0.2457
Purpose of having ultrasound	210/510 (41.2)	59/190 (31.1)	0.5931
Additional information would have liked to have WHILE having US			
Was there a problem	268/510 (52.5)	76/190 (40.0)	0.7198
Explanation of what the person who did the ultrasound saw	282/510 (55.3)	55/190 (28.9)	0.0026*
Other things planned to do after having US			
Talk to the baby’s father	454/510 (89.0)	154/190 (81.1)	0.8235
Talk to other family members about the findings	303/510 (59.4)	58/190 (30.5)	0.0104*

^a^Hypotheses test results: p-values calculated from Cochran-Mantel-Haenszel test (General Association statistic)

*Denotes p-value < 0.05

Of 700 women interviewed, 82% planned to attend the referral, while 73% actually attended. Barriers and motivations for attending referral visits are detailed below.

### Women who attended the referral visit (*N* = 510)

Of the 510 women who attended the referral visit, 68% said that attending the referral was easy. Facilitators included support from baby’s father (71%), short distance to the hospital (61%), clear referral instructions (52%), accessible transportation (36%), and support from family members/friends/neighbors (37%). Of these 510 women, 31% said attending the referral was difficult due to expense (77%), transportation (64%), and distance to hospital (42%).

### Women who did not attend the referral visit (*N* = 190)

Of the 190 women who did not attend the referral visit, 135 (71%) made no attempt due to expense (45%), lack of approval from the baby’s father (20%), transportation difficulties (16%), or distance to hospital (14%). Fifty-four women (28%) attempted going to the referral hospital but were unable to overcome barriers that included: being told to come back later (26%), being unable to ascertain where to go in the hospital (19%) and receiving no attention at the hospital (9%).

### Women’s understanding of why they were referred

We compared the sonographer’s reasons for referral with women’s recall of the findings (Table 5) to determine whether women understood why they were being referred. There was substantial agreement (AC1 > 0.90; excellent agreement between sonographers’ and women’s recall of ultrasound findings for possibility of miscarriage, ectopic pregnancy, incomplete miscarriage, fetal demise, multiple gestation, placenta previa, oligohydramnios and polyhydramnios and less agreement for malposition (AC1 statistic = 0.52; good agreement) and IUGR (AC1 statistic = 0.75; good agreement). When we separately examined the women who did and did not attend the referral visit, the agreement for IUGR was higher for women who attended the referral visit (AC1 statistic = 0.82; excellent agreement) compared to women who did not attend the referral visit (AC1 statistic = 0.52; good agreement).

**Table 5:** Ultrasound Exam Findings According to Sonographer and Women’s Recall by Referral Attendance Status

Ultrasound findings	Referral Status									
	Attended Referral, *N* = 510				Did not Attend Referral, *N* = 190					
	Positive Finding, *N* (%)		Agreement Coefficients		Positive Finding, *N* (%)		Agreement Coefficients		Overall Agreement Coefficients	
	Sonographer Findings	Woman’s Recall	% Agreement	AC1 Statistic	Sonographer Findings	Woman’s Recall	% Agreement	AC1 Statistic	% Agreement	AC1 Statistic
Possibility of miscarriage	1 (0.2)	9 (1.8)	98.0	0.98	1 (0.5)	2 (1.1)	99.5	0.99	98.4	0.98
Ectopic pregnancy	0 (0.0)	5 (1.0)	99.0	0.99	0 (0.0)	0 (0.0)	100.0	1.00	99.3	0.99
Incomplete miscarriage	0 (0.0)	1 (0.2)	99.8	1.00	0 (0.0)	0 (0.0)	100.0	1.00	99.9	1.00
Fetal demise	18 (3.5)	14 (2.7)	96.9	0.97	0 (0.0)	0 (0.0)	100.0	1.00	97.7	0.98
Fetal anomalies	13 (2.5)	69 (13.5)	87.1	0.85	6 (3.2)	5 (2.6)	97.4	0.97	89.9	0.88
Multiple gestation	82 (16.1)	69 (13.5)	93.1	0.91	6 (3.2)	8 (4.2)	97.9	0.98	94.4	0.93
Malposition	166 (32.5)	197 (38.6)	73.5	0.51	72 (37.9)	46 (24.2)	73.7	0.54	73.6	0.52
Placenta previa	32 (6.3)	25 (4.9)	93.5	0.93	9 (4.7)	6 (3.2)	96.3	0.96	94.3	0.94
IUGR (fetal growth restriction)	111 (21.8)	78 (15.3)	87.3	0.82	80 (42.1)	64 (33.7)	74.7	0.52	83.9	0.75
Oligohydramnios	31 (6.1)	33 (6.5)	94.9	0.94	26 (13.7)	23 (12.1)	90.0	0.87	93.6	0.92
Polyhydramnios	77 (15.1)	81 (15.9)	91.0	0.88	6 (3.2)	8 (4.2)	96.8	0.97	92.6	0.91
Other	48 (9.4)	39 (7.6)	87.3	0.85	14 (7.4)	37 (19.5)	76.3	0.69	84.3	0.81

*Values < 0.40 indicate poor agreement, 0.40–0.75 good agreement, and > 0.75 excellent agreement [15]

## Discussion

In this study, following an ANC visit that included ultrasound screening with a discovered complication, most women understood why they were being referred. The referred women who were provided a description of their next steps, including who they would see at the hospital, where to go, and what should happen, were more likely to attend their referral. We also found that sonographers can be trained to communicate the findings of the ultrasound examination to the women and that the women can understand the findings. Primary barriers to attending referrals, consistent with the literature, appeared to be related to cost, transportation, and distance. The need for approval of the baby’s father was both a facilitator for those that attended a referral visit and a barrier for those that did not. The additional barriers after reaching the hospital were also substantial reasons for not having a successful referral.

Few studies have been conducted in LMIC to understand pregnant women’s experiences with ANC ultrasound examinations. A study conducted in Ghana evaluated women’s experience and perception of ultrasound during ANC [21] and identified benefits and barriers. That study’s participants reported similar experiences and perceptions of ANC ultrasound as the participants in our study. Most of the Ghanaian study participants similarly perceived ANC ultrasound as useful and recognized the benefit of confirming fetal presentation and well-being. Several negative findings related to ultrasound examination were reported in the Ghanaian study, however, and are notable in their contrast to our findings. In the Ghanaian study, nearly half the women reported not being told the reason for the antenatal ultrasound examination and did not have the results explained to them. Less than one -quarter were invited by the provider to ask questions about the ultrasound examination findings, nor were the women shown the image of their baby on the monitor. Most Ghanaian participants indicated some level of dissatisfaction with the extent of the sonographer’s communication with them about the examination. Another study conducted in Kenya also found patients dissatisfied with the lack of communication from the provider, and it highlighted the reluctance of sonographers to answer questions [22]. A review article of obstetric ultrasound in LMIC discusses additional studies that found evidence of lack of communication between health providers and patients [14]. An Iranian study was described that found 48% of participants reported that the sonographer did not answer their questions and 90% reported that they were not shown an image of the fetus on the ultrasound screen. The authors wrote that women’s perceptions of and experience with ANC ultrasound are influenced by the quality of the communication between them and their health provider [14]. In contrast, 98% of First Look study participants indicated that the study sonographer informed them of what they saw on the monitor, 98% indicated that they received an image of the baby, 96% indicated that the sonographer showed them the images on the screen, and 98% found seeing the images helpful.

In our study, prior ultrasound experience among other mothers in the women’s social network was high, as was support for ultrasound among family members. Among both the women who did and did not attend the referral, a high proportion knew other mothers who had ultrasounds (89% vs 86%) and indicated that other family members thought that she should have an ultrasound (97% vs 94%). That women reported that the baby’s father thought she should have an ultrasound (96% for both groups) indicates a high level of acceptance of this technology in these rural health center settings.

In our sample, 73% of those who were referred attended the referral visit. This is consistent with the main study; of the 2233 women referred, 1589 (71%) attended a referral [16]. We observed a high level of understanding among women in both groups about the reasons why they were being referred, as demonstrated by high agreement between the sonographers’ documented findings and women’s recall of the ultrasound findings. This is likely attributed to the training that study sonographers received on how to communicate ultrasound findings to women, but could also be attributed to sonographers’ findings being confirmed or reinforced at referral or delivery.

### Strengths and limitations

A strength of our study is the large sample size: we completed high-quality structured interviews in a diverse sample of 700 women with possible pregnancy complications from five countries to identify motivators and barriers to following through with recommended referrals. Our sample thus comprised approximately one-third of the women identified with a possible pregnancy complication during the main study and provides a highly informative recounting of the healthcare experiences of women in several LMICs. We note that our results in this convenience sample could have biases, including the social desirability effect (the tendency of the participant to answer the survey in way they think the interviewer wants them to answer), selection bias (a possibility that women who participated in interviews differed from those who did not participate, in ways that were associated with their interview responses), and recall bias (possible systematic error in the participants’ recall of past experiences). These biases, if present, would lead to reduced generalizability of our findings to the source populations.

## Conclusion

The inclusion of ultrasound as a part of ANC is likely to continue increasing in rural low-resource settings. Research suggests that a central aim of its inclusion should be to encourage women with potentially complicated pregnancies to deliver in facilities providing a higher level of care [10, 11, 13–15]. The First Look study results emphasize that the inclusion of ultrasound screening by health center personnel is not by itself adequate to address the barriers women with complicated pregnancies face in delivering at referral hospitals. Important findings from this study suggest that better communication between the sonographer and the patient increase the likelihood of a successful referral, potentially identifying an intervention point in antenatal care processes. We also found substantial barriers to receiving care, even after reaching the hospital. Thus, there are at least three levels of communication needed to achieve completed referrals: transfer of ultrasound findings and associated information from the screening sonographer to the pregnant woman and the primary healthcare center staff, communication between the screening sonographer and the hospital, and a ready reception of the patient when she arrives at the hospital. Improvements in these communication pathways may facilitate the increased use of ultrasound screening results, with downstream effects on maternal and neonatal outcomes. Research to better understand and improve the efficacy of these communications is needed in order to obtain the maximum uptake and value from introducing ultrasound into ANC in community settings [23, 24].

## Abbreviations

ANC: Antenatal Care
DRC: Democratic Republic of Congo
LMIC: Low middle income countries
